# Lateral decubitus position vs. lithotomy position: which is the best way to minimize patient’s pain perception during transrectal prostate biopsy?

**DOI:** 10.1590/S1677-5538.IBJU.2015.0479

**Published:** 2017

**Authors:** Phil Hyun Song, Young Hwii Ko

**Affiliations:** 1 Department of Urology, College of Medicine, Yeungnam University, Daegu, Korea

**Keywords:** Pain Measurement, Patient Positioning, Biopsy

## Abstract

**Introduction:**

Considering the distinctive nature in terms of psychological stress and anal tone of position which is generally selected between lithotomy and left lateral decubitus (LLD), we postulated its effect on pain perception during biopsy, and investigated their association.

**Materials and Methods:**

A prospective study for comparison of two biopsy positions which were perform in a different working day was conducted for 208 men (lithotomy position=86, LLD=122). The decision on the position was made solely based on the patient’s preference for the biopsy day, and all procedures were performed according to the identical protocol (12-core biopsy with intrarectal lidocaine gel), probe, and needle. The maximal degree of pain during the entire process was assessed using a visual analogue scale (VAS), immediately after biopsy. After propensity matching, a total of 152 patients were finally selected (lithotomy group=76, LLD=76), then peri-biopsy parameters were compared.

**Results:**

Between groups, no differences were observed across all variables including age, obesity, prostate volume, serum PSA, international prostate symptom score, and cancer detection rate, except mean (±standard deviation) VAS score (3.89±2.01 vs. 4.58±2.22, p=0.049). VAS score showed significant association solely with patient’s position (Pearson’s coefficient=-0.165, p=0.042). In multiple linear regression models regarding the effect of clinical variables on VAS score, patient position was a single independent predictor favoring lithotomy position to decrease perceived pain (B=-0.928, p=0.024).

**Conclusions:**

These data suggest lithotomy position as a proper way to perform transrectal prostate biopsy with routine use of topical lidocaine gel in comparison with conventional LLD position.

## INTRODUCTION

In identification of prostate cancer, the most common malignant disease in Caucasians also with persistently increasing incidence in Asian populations, the use of extended number of biopsy cores became a contemporary standard in performance of transrectal ultrasound guided prostate biopsy (TRBx), by enhanced detection rate in comparison with a traditional six-core scheme (1, 2). After initial biopsy, the number of patients who requires repeat biopsy also grows with the increased life span and widespread use of active surveillance as a recommendable clinical option in management of low risk prostate cancer (3). In addition, along with increasing incidence of biopsy led by increased public awareness of the disease as well as escalation of biopsy cores obtained, the number of subjects who consider the procedure uncomfortable and painful is also increasing (4). Thus, efforts to minimize the discomfort associated with the procedure are crucial not only in detection, but also in a proper control of the disease through the patient’s life expectancy.

Although techniques including intrarectal lidocaine gel, periprostatic nerve block, and intravenous sedation with opioid drug, have been suggested to decrease the discomfort (5-7), the majority of patients still experience a considerable degree of pain (8). In an attempt to obtain a further relief, the effect of position which is generally selected between lithotomy and conventional left lateral decubitus (LLD) position had been researched (9-11). However, outcomes from several western studies reached contrasting conclusions, and there are no data for Asian population, who have a relatively smaller prostate. Considering the distinctive nature in terms of psychological stress, physician’s movement, and anal tone of each position, we postulated its effect on pain perception during the procedure, and investigated their association in Korean men in a prospective manner.

## MATERIAL AND METHODS

### The patients enrolled

Our indications for prostate biopsy were identical, including elevated serum prostate specific antigen (PSA) level over 3.5ng/mL and/or an abnormal digital rectal examination (DRE). In our institution, TRBx was performed in lithotomy position or LLD position and the decision on the position was made solely based on the patient’s preference for the biopsy date (lithotomy position on Monday and Friday; LLD position on Tuesday and Thursday). Biopsy in a lithotomy position was performed by a single urologist in the operative room and in a LLD position by a single radiologist in the radiologic department. At the initiation of this study, both physicians had over 10 years’ experience (minimal 500 cases each) in TRBx using identical position. Patients with anal and/or rectal pathologies, chronic pelvic pain syndrome, presence of urinary tract infection, or contraindication for the lithotomy position were excluded in this study. After approval of the local review board, 208 patients (122 subjects in LLD and 86 subjects in lithotomy position) were enrolled in this prospective study conducted from September 2013 to February 2014. Propensity score matching was then performed to control the imbalances between the groups, and 152 patients were finally selected for each group (76 subjects in LLD and 76 subjects in lithotomy position).

### Institutional transrectal prostate biopsy procedure

Entire procedures were performed according to the same protocol in terms of prophylactic antibiotics, local analgesics before the procedure, the number of biopsy cores, the model of needle and ultrasonography, and post procedural management regardless of the patient’s position. Anticoagulants or antiplatelets were routinely stopped for a minimum of 7 days before biopsy. Based on our institutional policy, all patients undergoing TRBx required hospitalization, and a cleansing enema was routinely performed prior to the day before the procedure. After overnight fasting following enema, the procedures were performed at a similar time (between 8-10AM). Ciprofloxacin 250mg was administered intravenously 1 hour before the procedure, and oral ciprofloxacin was prescribed for 3 additional days after the procedure. Immediately before biopsy, 10mL of 2% lidocaine gel was applied to the rectum for 5 minutes. To improve efficacy and reduce procedure time, TRBx was performed using a team based approach consisting of 4 participants, including a qualified physician who manipulated the probe and decided on the biopsy target area and depth, a senior resident with minimal 2 years’ experience assisting the procedure applying biopsy needles and obtaining the specimen, a junior resident who adjusted and maintained the patient’s position, and a scrub nurse who handled the specimen obtained from the biopsy needle. Specimens from 12 sites across the prostate (2 from the base, 2 from the mid lobe, 1 from the apex, and 1 from the transitional zone for each prostate lobe) were obtained using an 18-gauge 20-cm disposable needle (Baxter, USA), under the guidance of the same model of ultrasonography device (Hitachi HIGH VISION 5500; Hitachi Aloka Medical, Ltd, Tokyo, Japan) using the UST-675P prostate probe. When typical hypoechoic lesions suspicious of tumor were identified during procedure, additional biopsies were performed.

The highest degree of pain across the entire procedure from insertion of probe to completion of biopsy was assessed by a third person (non-physician coordinator) who was not participating in the procedure at the time of questioning, using a visual analogue scale (VAS) graded from 0 to 10 (0=painless, 10=intolerable pain), immediately after biopsy.

### Matching the patients and statistical analysis

Comparison of variables between lithotomy and LLD position was performed using chi-square test and Student’s T-test. Based on this comparison, the parameters that showed a statistically signiﬁcant difference between groups were selected, and then used for propensity score matching. Propensity scores were calculated for each patient using multivariable logistic regression.

The relationship between clinical variables and VAS was analyzed using simple correlation (Pearson’s correlation) and multivariable analysis using linear regression models. All statistical analyses were performed using SPSS, version 21.0 (SPSS Inc., Chicago, IL, USA) using two-sided tests with a significance level of 5%.

## RESULTS

### Basic demographics and matching the patient

The characteristics of the patients are summarized in Table-1. Before matching, the patients in lithotomy position had significantly severe lower urinary tract symptoms, which was assessed using international prostate symptom score (IPSS; 15.49±6.19 vs. 13.24±9.11, p=0.042) and marginally higher pre-biopsy PSA (22.76±27.50ng/dL vs. 16.01±18.81ng/dL, p=0.005). Propensity score matching was then performed for 4 pre-biopsy variables, including IPSS, PSA, prostate volume, and age, considering previously reported links between the last two variables for the first two variables. For finally selected 152 patients, statistical similarity was obtained for all pre-biopsy variables (Table-1).

**Table 1:** Characteristics of the enrolled patients.

		Before matching (n=208)				After matching (n=152)			

		Total	LLD position (n=122)	lithotomy position (n=86)	p-value	Total	LLD position (n=76)	lithotomy position (n=76)	p-value
Pre-biopsy variables	**Age (years)**	67.81±8.33	67.73±8.881	67.93±7.539	0.861	67.16±8.45	66.72±9.19	67.59±7.67	0.528
	**Prostate volume (g)**	42.17±25.86	40.33±20.25	44.78±32.14	0.258	42.84±27.86	40.56±21.14	45.12±33.24	0.315
	**Pre-biopsy PSA (ng/dL)**	22.99±18.8	16.01±18.81	22.76±27.50	0.050	18.77±22.87	16.11±17.88	21.44±26.82	0.151
	**Number of biopsy (%)**								
	first	180 (86.5)	103 (84.4)	77 (89.5)	0.456	135 (88.8)	67 (88.2)	68 (89.5)	0.763
	second	21 (10.1)	15 (12.3)	6 (7.0)		12 (7.9)	7 (9.2)	5 (6.6)	
	third	7 (3.4)	4 (3.3)	3 (3.5)		5 (3.3)	2 (2.6)	3 (3.9)	
	**Nodule on DRE (%)**								
	with palpable nodule	9 (5.4)	5 (5.3)	4 (5.4)	0.620	6 (4.9)	2 (3.4)	4 (6.3)	0.682
	without palpable nodule	159 (94.6)	89 (94.7)	70 (94.6)		116 (95.1)	56 (96.6)	60 (93.8)	
	**BMI (kg/m** ^**2**^ **)**	23.82±2.67	23.71±2.57	23.97±2.77	0.485	23.86±2.60	23.92±2.45	23.79±2.76	0.754
	**Prior history of DM (%**)								
	with DM	36 (17.3)	20 (16.4)	16 (18.6)	0.712	31 (20.4)	16 (21.1)	15 (19.7)	1.000
	without DM	172 (82.7)	102 (83.6)	70 (81.4)		121 (79.6)	60 (78.9)	61 (80.3)	
	**Pyuria at time of biopsy (%)**				0.601				
	with pyuria	10 (4.8)	6 (4.9)	4 (4.7)		6 (3.9)	4 (5.3)	2 (2.6)	0.681
	without pyuria	198 (95.2)	116 (95.1)	82 (95.3)		146 (96.1)	72 (94.7)	74 (97.4)	

	**Total IPSS**	14.12±8.15	13,24±9.11	15.49±6.19	0.042	15.28±7.94	15.07±9.40	15.49±6.19	0.745

Intra & post - biopsy variables	**VAS score**	4.29±2.20	4.54±2.28	3.89±2.02	0.041	4.24±2.14	4.58±2.22	3.89±2.01	0.049
	0	4 (2.1)	4 (3.4)	-	0.105	3 (2.0)	3 (3.9)	-	0.157
	1	11(5.6)	4 (3.4)	7 (9.2)		10 (6.6)	3 (3.9)	7 (9.2)	
	2	37 (19.0)	19 (16.0)	18 (23.7)		27 (17.8)	9 (11.8)	18 (23.7)	
	3	22 (11.3)	11 (9.2)	11 (14.5)		19 (12.5)	8 (10.5)	11 (14.5)	
	4	38 (19.5)	24 (20.2)	14 (18.4)		30 (19.7)	16 (21.1)	14 (18.4)	
	5	19 (9.7)	15 (12.6)	4 (5.3)		11 (7.2)	7 (9.2)	4 (5.3)	
	6	37 (19.0)	23 (19.3)	14 (18.4)		32 (21.1)	18 (23.7)	14 (18.4)	
	7	8 (4.1)	4 (3.4)	4 (5.3)		6 (3.9)	2 (2.6)	4 (5.3)	
	8	15 (7.7)	11 (9.2)	4 (5.3)		13 (8.6)	9 (11.8)	4 (5.3)	
	9	-	-	-		-	-	-	
	10	4 (2.1)	4 (3.4)	-		1 (0.7)	1 (1.3)	-	
	**Number of biopsy core**	12.13±0.627	12.02±0.589	12.29±0.648	0.006	12.19±0.524	12.08±0.271	12.30±0.673	0.009
	12 core (%)	179 (86.1)	112 (91.8)	67 (77.9)		129 (84.9)	70 (92.1)	59 (77.6)	0.022
	over 12 core (13-16%)	29 (13.9)	10 (8.2)	19 (22.1)	0.007	23 (15.2)	6 (7.9)	17 (22.4)	
	**Pca detected (%)**	85 (40.9)	54 (44.3)	31 (36.0)	0.254	56 (36.8)	29 (38.2)	27 (35.5)	0.867

**SD** = standard deviation; **BMI** = body mass index; **PSA** = prostate-specific antigen; **IPSS** = International prostate symptom score; **VAS** = visual analogue scale

After biopsy, the overall cancer detection rate was 36.8%, which was similar across each group (p=0.867) despite of significantly more biopsy cores were obtained in lithotomy position (12.30±0.673 vs. 12.08±0.271, p=0.009). During the procedure, while no differences were observed in overall distribution of VAS and equinoctial distribution using cutoff score of 5 (p=0.157 and 0.099), the mean value of VAS was significantly lower in the lithotomy position group (3.89±2.01 vs. 4.58±2.22, p=0.049), when it was treated as continuous variables.

### Clinical variables associated with VAS

In simple correlation analysis, VAS score showed significant association solely with patient’s position preferring lithotomy position to decrease perceived pain (Pearson’s coefficient=-0.165, p=0.042, Table-2). In multiple linear regression models (stepwise method, R^2^=0.042, p=0.024) regarding the effect of clinical variables on VAS score, patient position was a single independent predictor (B=-0.928, p=0.024, Table-3) favoring lithotomy position.

**Table 2:** Outcome of simple correlation among clinical variables associated with VAS. correlation among clinical variables associated with VAS.

Variables	Pearson’s coefficeint	p-value
**Age**	-0.068	0.402
**Prostate volume**	0.005	0.954
**Pre-biopsy PSA (ng/dL)**	-0.003	0.974
**Number of biopsy**	0.067	0.410
**Number of biopsy core**	-0.036	0.661
**The presence of nodule on DRE**	-0.176	0.053
**BMI (kg/m** ^**2**^ **)**	-0.060	0.463
**Prior history of DM**	0.041	0.620
**Pyuria at time of biopsy**	-0.015	0.856
**Total IPSS**	-0.117	0.152
**Identification of Pca**	-0.015	0.852
**Position at the time of biopsy**	-0.165	0.042

**PSA** = prostate-specific antigen; **DRE** = Digital rectal examination; **BMI** = body mass index; **DM** = Diabetes mellitus; **Pca** = Prostate cancer

**Table 3:** Outcome of multiple linear regression model among clinical variables associated with VAS.

Variables	P value	B (95% CI)
**Age**	0.199	-0.115
**Prostate volume**	0.071	-0.161
**Pre-biopsy PSA (ng/dL)**	0.546	0.055
**Number of biopsy**	0.336	0.086
**Number of biopsy core**	0.655	-0.041
**The presence of nodule on DRE**	0.247	-0.104
**BMI (kg/m** ^**2**^ **)**	0.272	-0.098
**Prior history of DM**	0.827	-0.020
**Pyuria at time of biopsy**	0.746	-0.029
**Total IPSS**	0.613	-0.045
**Identification of Pca**	0.434	-0.070
**Position at the time of biopsy**	0.024	-0.928 (-0.119~ -1.644)

**PSA =** prostate-specific antigen; **DRE =** Digital rectal examination; **BMI =** body mass index; **DM =** Diabetes mellitus; **Pca =** Prostate cancer

## DISCUSSION

TRBx procedure was generally believed to be well tolerable for the majority of patients (11). However, contrary to the traditional perception by the urologist, the pain or discomfort of patients associated with biopsy is not mild or negligible. Some kind of discomfort or pain during the procedure is reported by 52-96% of patients and 20% of them suffer from severe pain (12, 13). Even by DRE alone, 73% of patients reported moderate or higher discomfort (14). A clear tendency between the degree of pain during biopsy and the number of biopsy cores was consistently reported by prospective trials (15, 16). Regardless of the number of biopsy cores, the incidence of severe pain score increased generally from the first to the last biopsy (15). Tolerance of biopsy remained unchanged throughout the procedure in 53.2% and became worse as the test proceeded in the remaining patients (17). Familiarity with the procedure at the repeat biopsy did not decrease pain or anxiety at al. (18).

In contrast, only 4% and 11% of patients reported no pain or discomfort, respectively, and 3% had no complaint during TRBx (18). Of men who were interviewed, 19% would not wish to undergo the procedure again without aid of analgesia, and 6% would like biopsies to be done under general anesthesia (19). Because the biopsy itself is still invasive in nature, a high degree of discomfort associated with it may result in failure of the patient to return for the future biopsy even though it will be necessary.

Perception of pain is a highly subjective psychological phenomenon, which can be influenced by various factors. As for the predictor of severe pain during TRBx, several reports have suggested preoperative anxiety, which was reported in 64% of biopsy events (18), pain on insertion of the transrectal ultrasonic probe (15) or during DRE (20), and age of the patients (19). However, all of these previously reported characteristics were not chosen or adjustable by the physician, without providing a substantial clue to minimize the discomfort at the time of the procedure despite usefulness in identification of the risk group. Conversely, the outcomes from this series suggest lithotomy position as a simple method for the majority of subjects who have no limitation in range of motion in the hip joint. A significant decrease of mean VAS score was observed in lithotomy position in comparison with LLP, and a multivariable model showed lithotomy position as a single significant predictor to minimize VAS score.

Then, what is the potential explanation for our findings? While the mechanism of pain associated with TRBx is complex, recent studies have gradually enlightened this area. Between lithotomy position and LLP, there exist two fundamental differences; the visibility of the procedure by the subject or eye contact by the physician which thereby influences the embarrassment of the subject, and the convenience in relaxation of pelvic floor muscle which affects the anal sphincter contraction thereby enabling easier probe insertion and lesser pain perception. Because a sense of vulnerability or defenselessness associated with patient positioning may interfere with physical and psychological distress (14), lithotomy position which allows the patient to identify visual information on the progress of the procedure improves the tolerance of the patient while the position of the legs in this position obviously creates additional discomfort. A more direct relationship between patient’s positioning and the degrees of pelvic floor muscle relaxation was recently identified. Using electromyographic evaluation with eight-channels for 29 women, Resende et al. demonstrated that the lateral positon presented a significantly greater myoelectrical signal of pelvic floor resting tone among lithotomy, supine, and lateral positions (21). In the same context, several randomized controlled trails which assessed the effect of topical muscle relaxant during TRBx consistently confirmed the effectiveness and safety in diminishing the patient’s discomfort, particularly during the insertion of an ultrasound probe (22, 23).

Our hypothesis regarding the positive influence of lithotomy position on TRBx was supported by other research, which demonstrated that use of a larger probe (74mm) results in much higher VAS pain perception than same size and smaller (58mm) probe in the absence of injectable local anesthesia (24). In addition, probe insertion was reported to produce a significantly higher pain scale than biopsy using a 12 core prostate biopsy scheme (25). Due to the similarities of the procedure, discomfort during DRE can reflect that of the patient during TRBx, and several studies have reported an association between the patient’s position and pain during DRE (20, 26). Among four positions including LLD and supine position, more than half of their patients chose the supine position for DRE (27).

The authors also recognize several limitations of this series. First, while the data were collected prospectively, we cannot randomize the subjects based only on the position, mainly because of uneven distribution of patient’s preference on the date. Instead, we adjusted the discrepancies of each group by propensity matching, and selected subjects demonstrated similar pre-biopsy characteristics across all variables except the number of biopsy core, which was rather significantly higher in lithotomy position. However, the number of biopsy core was not significantly associated with VAS both in univariable and multivariable analysis. Second, despite similar expertise of each physician on the procedure, there may exist a habitual difference which may affect the pain perception of the patients. In addition, the environmental difference of OR and radiologic department may act as an isolated variable. Third, different from the majority of reported series, our procedure was performed after hospitalization. Our institutional policy, based on prior reports on discomfort and complications related to the TRBx, requires hospitalization which facilitates the routine use of enema preparation before the procedure and detailed counselling from the coordinator or physician, both of which may have a positive effect on VAS score. Thus, prostate biopsy in different settings may not lead to reproduction of a similar result with us, and we believe these distinctive natures as a main reason for inconsistent conclusions on the advantage of lithotomy position during TRBx in prior series which used different biopsy setting in terms of the use of analgesics, the number of biopsy cores, and the number and experiences of physicians (9-11).

It is also obvious that not all men experience severe endurable pain during the procedure. However, in performing prostate biopsy, one of the goals should be to minimize the patient’s discomfort associated with the procedure in era of active surveillance strategy in which the acceptance of repeat biopsy is crucial. In acquisition of this, our data indicating an obvious influence of position on pain may contribute to establishment of the best clinical setting for TRBx.

## CONCLUSIONS

The position of the patient was a single factor associated with pain perception during transrectal prostate biopsy with an extended biopsy scheme. With routine use of topical lidocaine gel, lithotomy position significantly decreased the patient’s pain without compromising detectability of prostate cancer. Based on these findings, we suggest lithotomy position as a proper way to perform TRUS guided prostate biopsy in comparison with conventional lateral decubitus position.
